# 3D microfluidics-assisted modeling of glucose transport in placental malaria

**DOI:** 10.1038/s41598-022-19422-y

**Published:** 2022-09-10

**Authors:** Babak Mosavati, Andrew Oleinikov, E. Du

**Affiliations:** 1 Department of Ocean and Mechanical Engineering, Florida Atlantic University, Boca Raton, FL 33431 USA; 2 Department of Biomedical Science, Charles E. Schmidt College of Medicine, Florida Atlantic University, Boca Raton, FL 33431 USA

**Keywords:** Biotechnology, Biophysics

## Abstract

The human placenta is a critical organ, mediating the exchange of nutrients, oxygen, and waste products between fetus and mother. Placental malaria (PM) resulted from *Plasmodium falciparum* infections causes up to 200 thousand newborn deaths annually, mainly due to low birth weight, as well as 10 thousand mother deaths. In this work, a placenta-on-a-chip model is developed to mimic the nutrient exchange between the fetus and mother under the influence of PM. In this model, trophoblasts cells (facing infected or uninfected blood simulating maternal blood and termed “trophoblast side”) and human umbilical vein endothelial cells (facing uninfected blood simulating fetal blood and termed “endothelial” side) are cultured on the opposite sides of an extracellular matrix gel in a compartmental microfluidic system, forming a physiological barrier between the co-flow tubular structure to mimic a simplified maternal–fetal interface in placental villi. The influences of infected erythrocytes (IEs) sequestration through cytoadhesion to chondroitin sulfate A (CSA) expressed on the surface of trophoblast cells, a critical feature of PM, on glucose transfer efficiency across the placental barrier was studied. To create glucose gradients across the barrier, uninfected erythrocyte or IE suspension with a higher glucose concentration was introduced into the “trophoblast side” and a culture medium with lower glucose concentration was introduced into the “endothelial side”. The glucose levels in the endothelial channel in response to CSA-adherent erythrocytes infected with CS2 line of parasites in trophoblast channel under flow conditions was monitored. Uninfected erythrocytes served as a negative control. The results demonstrated that CSA-binding IEs added resistance to the simulated placental barrier for glucose perfusion and decreased the glucose transfer across this barrier. The results of this study can be used for better understanding of PM pathology and development of models useful in studying potential treatment of PM.

## Introduction

Placenta plays a vital role in fetal development by providing the fetus with nutrients and oxygen and removal of their waste products^1^. Malaria infections by *P. falciparum* in pregnancy, which called placental malaria (PM), causes severe adverse outcomes such as low birth weight (LBW)^2^, intrauterine growth restriction^3^, premature birth^4^, or abortion^5^. PM contributes to around 200,000 newborn deaths annually^6^. In PM, sequestration of infected erythrocytes (IEs) at the placental syncytiotrophoblast is CSA specific^7,8^, mediated by the infected erythrocytes surface-expressed malarial PfEMP1 protein VAR2CSA^9^. Sequestered IEs may alter the physiological functions of syncytiotrophoblast and consequently affect the fetal growth and development^10^. Often, sequestration of IE in the placenta stimulates circulating monocytes and tissue-resident macrophages to infiltrate and accumulate in the intervillous space. This, in turn, induces a local inflammation, which is a major contributor to the severity of the disease and the consequential pathologies including intrauterine growth restriction and LBW, a leading cause of newborn mortality^10,11^. The mechanisms underlying these pathologies are still incompletely characterized, but may include placental insufficiency due to poor placental development^12^. Studying the molecular transport between maternal and fetal compartments may help to understand some of the pathophysiological mechanisms in PM.

The placenta is a temporary organ that develops during pregnancy and its structure can change over the gestational period. Clearly, studying placental pathology in vivo, including PM, is possible, but difficult due to multiple reasons. Therefore, different models in vivo^13,14^, ex vivo^15,16^, and in vitro^17,18^ have been developed, including various models to study transport across the placental barrier. Due to some differences in placenta anatomy in animals and humans, there are some challenges for in vivo studies using animal models. The ex vivo models do not cause any severe damage to the maternal vasculature^19^, but they are limited by challenges in obtaining permission from women to participate in investigations and by complexity of the maternofetal barrier structure. In several studies, animal models have been applied to investigate the pathogenesis of PM. Hviid et al.^20^ recently reviewed different rodent models of PM. As rodent malaria parasites do not possess the family of erythrocyte membrane protein 1 (PfEMP1), the results of these studies remain controversial. Placentas donated by pregnant women have been used as ex vivo models^21^. Clearly, PM may produce pathophysiological effects through a number of pathways and mechanisms. We are specifically interested in modeling effects of IE cytoadhesion on transport of molecules across the placental barrier. In this respect, Moro et al.^22^ studied the transfer of antimalarial antibodies from pregnant women to their fetuses. They demonstrated that transfer of antibodies is reduced in pregnant women with malaria. Lybbert et al.^23^ studied the abundance of megalin and its intracellular adaptor protein Dab2 in placental malaria. They demonstrated that placental infection in malaria is associated with reduced abundance of megalin transport/signaling system, which may contribute to low birth weight, a primary cause for infant mortality. We believe that development of various in vitro models of placenta, including placenta-on-a-chip, will allow better understanding of a multitude of processes induced by sequestration of IE to placental syncytiotrophoblast in PM at the molecular and cellular levels.

The engineered microchips that can recapitulate not only the significant elements of microscopic architecture but also the biophysiological characteristics of human placenta have been developed. These microchips, known as placenta-on-a-chip devices, serve as a platform in support of formation of microengineered placental barriers through culturing and arrangement of essential cell types, management of cellular and subcellular environments, as well as mimicking blood circulations. These placenta-on-a-chip devices have shown great potential to be alternative approaches to traditional animal models for drug testing and screening^24^. Blundull et al.^25^ developed a model to study the glyburide diffusion across the human placental barrier. Their results demonstrated that glyburide transport rate was similar in their model and ex vivo human placenta techniques. Yin et al.^26^ developed a 3D placental barrier-on-a-chip microdevice to study the environmental exposure to nanoparticles. Mosavati et al.^27^ developed a 3D placental interface model to analyze the glucose diffusion across the simulated placental barrier under shear flow conditions experimentally and numerically. Miura et al.^28^ studied the molecular mechanism underlying the microvillie formation induced by fluid shear forces in the placental barrier microdevice. See references for comprehensive reviews on placenta-on-a-chip models and their applications in studying placental diseases^29^ and drug transport^30^. Despite the advances in biosensing and live cell imaging, interpretation of transport across the placental barrier remains challenging, since placental nutrient transport is a complex problem involving multiple cell types, multi-layer structures, as well as coupling between cell consumption and diffusion across the placental barrier.

The anatomy of human placenta and architecture of maternal–fetal interface, i.e., interface between maternal and fetal blood, are complex and cannot be easily reconstructed in their entirety in the modern in vitro models. Most of the molecular exchange between maternal and fetal blood occurs in the branching tree-like structures called villous trees^31^. Because Placental Malaria (PM) may start only after beginning of second trimester when intervillous space opens to IE and monocytes^32^, we were interested in the placental model of maternal–fetal interface formed in the second half of pregnancy. There are several layers of cells and membranes that separate the maternal blood, which brings nutrition and molecular signals to and removes various molecules from the developing fetus, from the fetal blood. These include^33^ syncytiotrophoblast, a multinucleated cell with a surface area of ~ 10–11 m^2^ covering the surface of the floating villi, and underlying cytotrophoblasts that syncytialize to contribute to the expansion of syncytiotrophoblast throughout pregnancy^34^; fused basal lamina; and capillary endothelium that faces fetal blood. This is a complex structure that can be simulated using in vitro models to some extent. Therefore, in this work our simplified model was composed of three layers: BeWo cells simulating “trophoblast side” of the placental villi facing maternal blood, HUVEC cells simulating “endothelial side” of the villi facing fetal blood, and a porous collagen membrane separating the BeWo layer (surrogate of cytotrophoblasts) from the endothelium. This model was aimed at simulating glucose transport across the placental barrier in the presence and absence of CSA-binding *P. falciparum* IE (CS2 line) which sequester to BeWo cells through interactions with CSA molecules—the events that take place during early PM, before the influx of monocyte/macrophages in the later stages of PM. This influx may further exacerbate the pathophysiology of this severe malaria complication and might be investigated using this model in future work.

In this paper, we describe the development of a 3D microfluidics-based model of the placental barrier using the 3-lane organo-plate microdevice. This microdevice enables us to measure the glucose diffusion across the modeled placental barrier and the effects of blood infection with CSA-binding *P. falciparum* line. Co-culturing of the BeWo cells and human umbilical vein endothelial cells (HUVECs) on the contralateral sides of the ECM gel was used to mimic the physiological placental barrier in vitro. The comparison between the glucose transport rate across the placental barrier in conditions when uninfected or *P. falciparum* infected blood flows on trophoblast side helps to better understand this important aspect of PM pathology and potentially might be used as a model to study PM treatment. Theoretical model of biological mass transport is utilized to dissect the mechanisms of transport across placental barrier characterized by interference of cell consumption and diffusion/transport potentially impeded by IE sequestration that imitate events during PM. This study provides novel information on nutrition exchange affected by *P. falciparum* infected erythrocytes and potentially this or similar microdevice may serve as a model for other placenta-relevant diseases.

## Materials and methods

### BeWo cells, HUVECs and *Plasmodium falciparum* cultures

Human umbilical vein endothelial cells (HUVECs; ATCC® CRL-1730.) were cultured in endothelial cell growth medium kit (EGM-2; Lonza, Alpharetta, GA, USA, Cat. No. CC-3162). Human trophoblast cell line (BeWo cells; ATCC® CCL-98) was cultured in Ham’s F-12K nutrient mixture (Corning Cat. No. 10-025-CV) with 10% fetal bovine serum (ATCC Cat. No. 30-2020) and 40 mg/mL gentamicin (GIBCO, Gaithersburg, MD, USA, Ref. No. 15-750-060). The HUVEC and BeWo cells were cultured in humidified incubator at 37 °C in 5% CO_2_ atmosphere to reach 90% confluence. The CS2 strain of *P. falciparum* that binds to CSA was grown and enriched following protocols published in previous research^35^. Briefly, the CS2 parasite line was grown in human O+ erythrocytes at 2% hematocrit in complete RPMI 1640 medium supplemented with 40 µg/mL gentamicin sulfate and 10% human serum for about 3–5 growth cycles using a gas mixture of 90% N_2_, 5% CO_2_ and 5% O_2_ at 37 °C. Mature cytoadhesive forms of infected erythrocytes (IE) were enriched by magnetic LD columns (Milteni Biotec, cat#130-042-901) as described by the manufacturer. The parasitemia value of the infected erythrocyte cells ranged from 26 to 95% in various preparations that have been used in our experiments.

### Microfluidic modeling of placental barrier

**Figure 1:**
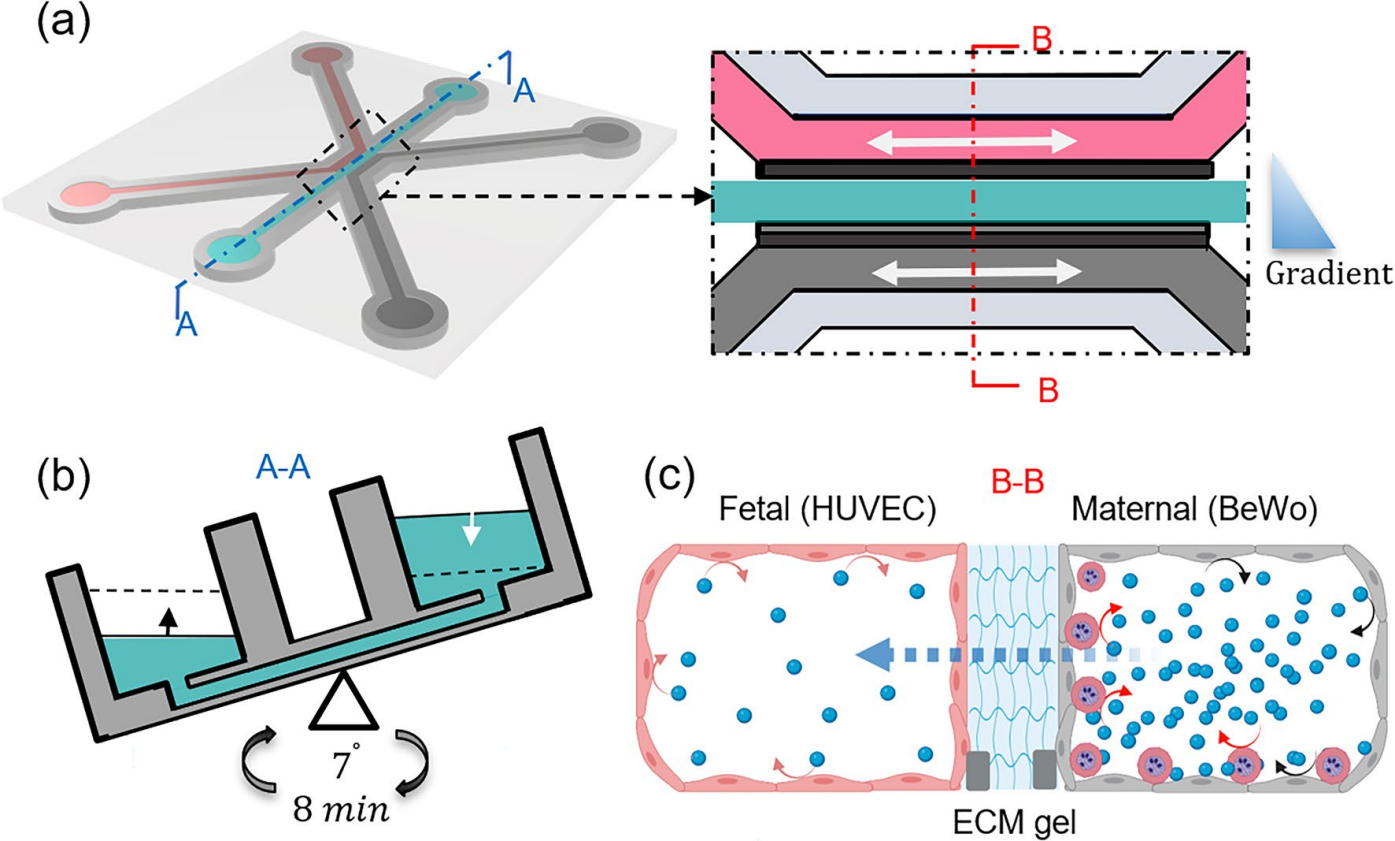
(**a**) Overview of the method for modeling placenta malaria in the 3-lane OrganoPlate platform. (**b**) Flow generation in the microfluidic channels by rocking the OrganoPlate between a + 7° and − 7° inclination every 8 min. (**c**) Schematic of the placental barrier model showing the glucose (blue dots) transport (dashed arrow) and cellular consumption (curved arrows) by BeWo cells, HUVECs and IEs. Created with BioRender.com.

The 3-lane OrganoPlate (MIMETAS No. 4003-400-B, Leiden, The Netherlands) was used to develop the placental barrier model. It consists of microfluidic chips in a microtiter plate format. Each microfluidic chip consists of three channels, a center channel for loading an extracellular matrix (ECM), side by side perfusion channels for HUVECs and BeWo cell cultures (Fig. 1). Each channel is 300 µm wide and 220 µm high and connected to two medium reservoirs. To prepare the ECM gel, 4 mg/ml Type I collagen (Collagen I Rat Tail, Advanced Biomatrix, USA, Cat. No. 5153), 100 mM HEPES (Life Technologies, Cat. No. 15630106), 3.7 mg/ml NaHCO3 (Sigma, Cat. No. S5761) were mixed in a 1:1:8 ratio. To avoid generating bubbles, these materials were thoroughly mixed by gently pipetting the mixture up and down (> 20 times) while keeping it on ice. 2 µl of ECM gel was injected into the middle inlet. After that, the OrganoPlate was placed in the humidified incubator for 30 min to allow the polymerization of the collagen-I gel. Then 30 µl phosphate buffered saline (PBS) was injected into the middle inlet to prevent the gel from drying out. A suspension of BeWo cells (10,000 cells per µl) was injected to the trophoblast channel (maternal circulation) after removing the PBS from the channel. The OrganoPlate was placed on its side in the MIMETAS plate in a CO_2_ incubator for 3 h, allowing cells to attach. Next, a suspension of HUVEC cells (10,000 cells per µl) was injected to the endothelial channel (fetal circulation). HUVECs were attached to the opposite side of ECM after placing the OrganoPlate in the MIMETAS plate for 3 h. During this procedure, cells can attach to all interior surfaces of the channel.

After the microfluidic channels were seeded with cells, the reservoirs of each channel were filled with identical volume (50 µL) of corresponding culture media. Then, the OrganoPlate was placed in a CO_2_ incubator on an interval rocking platform (OrganoFlow, MIMETAS) switching between + 7° and − 7° inclination every 8 min (Fig. 1b). The rocking motion was used to create a bidirectional flow during cell culture, facilitating a uniform lining of the cells inside the microfluidic channels under shear stresses of 0–1.41 dynes/cm^2^ according to the manufacturers’ specifications.

Cell culture media was refreshed daily by aspirating and replacing the medium from medium inlets and outlets for each cell type. Delivery of the culture media was achieved by the rocking motion that circulated the media back and forth between each two reservoirs. Confluent cell layers were obtained after 10 days of culture and became ready for blood perfusion experiments. In this study, normal and PM blood flow conditions were modeled by introducing uninfected erythrocytes and CS2 IEs into the trophoblast channels, respectively. A volume of 10 µl sample of erythrocytes (5.5 × 10^6^–6.9 × 10^6^ cells/ml) suspended in RPMI 1640 were injected into the trophoblast channels. Then the Organoplate was inclined at 45° and placed in a CO_2_ incubator for 3 h to allow all erythrocytes (uninfected and CS2 IEs) to settle and interact with BeWo cells by gravity. Afterwards, the unattached erythrocytes were removed from the trophoblast channels and these channels were washed with PBS three times by gently pipetting 20 $\mathrm{\mu l}$ PBS each time. The trophoblast channels were filled with F12K Medium before starting the glucose transport experiment.

### Image acquisition and analysis

Cells were stained with calcein-AM and nuclei stained with Hoechst 33,342 for imaging by a LSM 700 laser scanning confocal microscope using 40X Plan Apo objective and a 60 µm pinhole spinning disk. A z-series of confocal sections of the cellular microfluidic channels was gathered at an imaging depth of 12 bits and the frame size of 1024 × 1024, from which 3D images were rendered using the ImageJ/Fiji^36^. The nuclei of both cell types were stained with Hoechst 33,342 (Fisher Scientific, Carlsbad, CA, USA, Cat. No. H3570).

### Measurement of glucose transfer across the placental barrier

To study the effects of CS2 sequestration on the glucose transfer across the placental barrier, an initial glucose gradient was created within the physiological values^37^. Specifically, the F12-K culture Medium with a higher glucose concentration of 159 mg/dl and EGM-2 medium with lower glucose concentration of 97 mg/dl were introduced into the trophoblast and endothelial channels, respectively. The medium perfusion was maintained by placing the OrganoPlate on the OrganoFlow switching between a + 7° and − 7° inclination every 8 min for 2 h. Finally, samples were collected from inlet and outlet of each channel and measured using a GM 100 glucose meter (BioReactor Sciences, Lawrenceville, Georgia, USA). The glucose concentration in each channel was averaged based on triplicate measurements of its inlet and outlet. Analysis of glucose transfer was based on 9 and 11 microdevices for normal and PM conditions, respectively.

### Statistical analysis

Experimental data were shown as mean ± standard error of the mean (SEM), or whisker-box plots (box, 25–75 percentile range; whiskers, 5–95 percentile range; horizontal line, median). Two sample t-test was used to compare between control and experimental groups. The values of *p < 0.05 are considered statistically significant.

## Results and discussion

### Placental barrier model

BeWo cell line is the most extensively used in vitro model of various placental functions*.* It reveals most of the characteristics of cytotrophoblast cells, including expression of normal receptors and syncytial fusion^38,39^. They have been used in other works on modeling materno-fetal barrier and placenta-on-a-chip^40–42^. Syncytial fusion can be further stimulated by treatment with forskolin but even without it BeWo cells can syncytialize^38,39^. In this two cell-layers model (BeWo and endothelial cells (HUVEC) on the other side of membrane) we decided not to use forskolin for several reasons. First, as we mentioned, BeWo cells have some level of syncytialization even without forskolin. Second, non-syncytialized cells might be considered as relevant cytotrophoblast cells that in vivo located under the syncytiotrophoblast. As our model cannot reproduce these features of placenta and is a simplified model, in this case it represents a mixture of two types of relevant cells on the trophoblast side of the placenta, though not in multilayer arrangement. Third, and important, we grew two types of cells at the same time, and we did not want to treat EC with forskolin, as it significantly increases cAMP levels in treated cells^43^, which may produce some unexpected effects. Without this treatment we would have some level of syncytialization of BeWo cells and avoid potential effects of forskolin treatment on HUVEC. Therefore, one of the limitations of this work is that this model does not contain a single syncytiotrophoblast layer facing simulated “maternal” blood and does not contain some number of cytotrophoblast under this layer.

**Figure 2:**
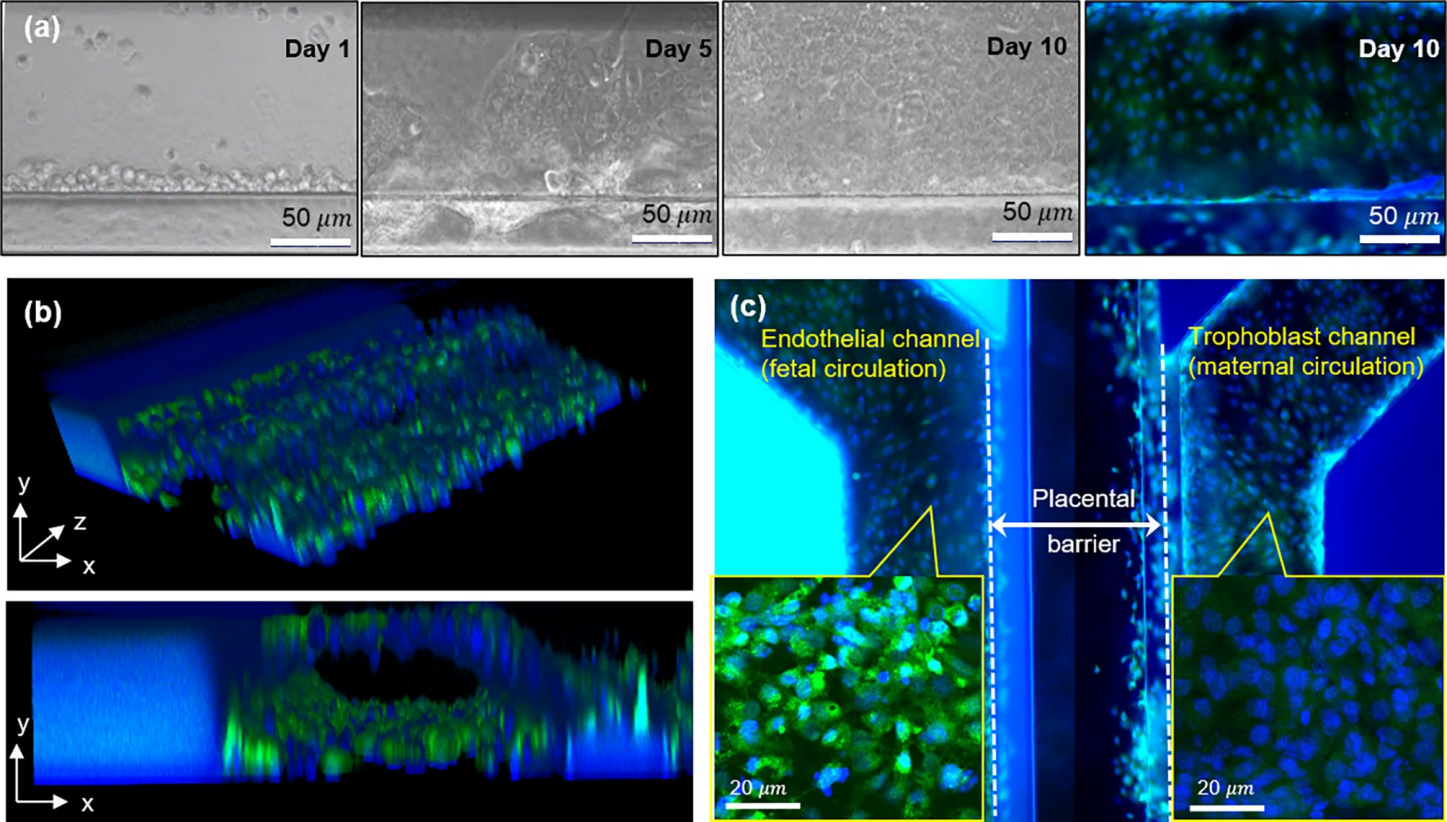
Modeling of placental barrier for permeability experiments, by co-culturing BeWo/HUVECs on the contralateral sides of ECM gel in the 3-lane OrganoPlate. (**a**) Formation of BeWo monolayer in the trophoblast channel throughout 10 days. (**b**) Entire inner surface of the trophoblast channel being covered by BeWo cells after 10 days. (**c**) Placental barrier model ready for perfusion experiment. Image of HUVECs and BeWo cells stained with calcein-AM and nuclei stained with Hoechst 33,342.

Barrier permeability model was achieved after 10 days of 3D cell co-culture in the 3-lane OrganoPlate. Figure 2a shows the progress of BeWo cell proliferation over time in the trophoblast channel, at day 1, 5 and 10, respectively. Figure 2b shows the 3D reconstruction of trophoblast channel stained with calcein and Hoechst 33,342, where the BeWo lining resulted in the entire inner surface of the trophoblast channel being covered with cells after 10 days. Figure 2c shows a representative stitched image for the placental barrier between the trophoblast and endothelial channels.

### IE sequestration

**Figure 3:**
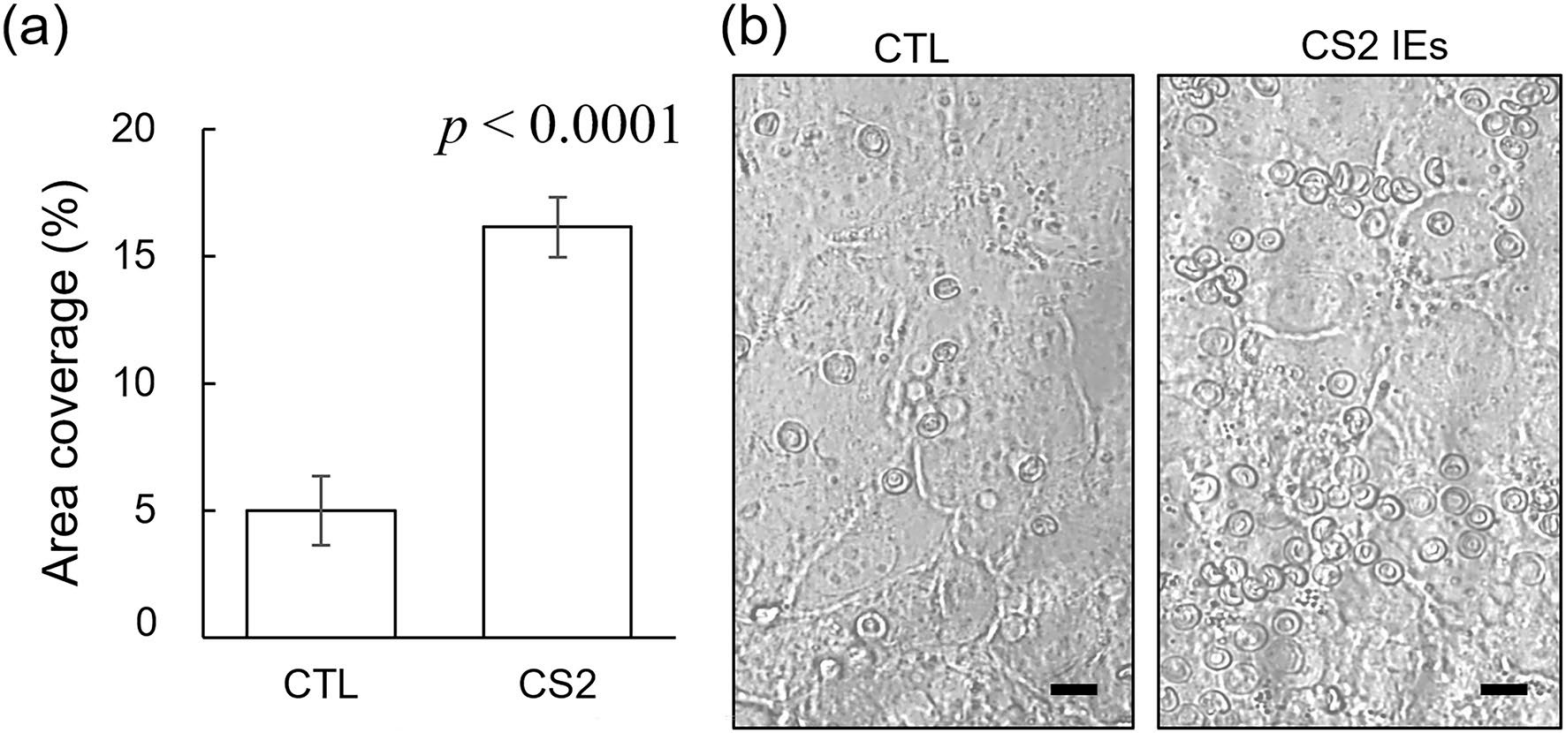
IE sequestration in placental barrier. (**a**) Adhesion of IEs and uninfected erythrocytes (CTL) to the placental barrier, as quantified by the percent of area coverage by erythrocytes. Errors bars show 1 SD. Data represent measurements made in 3 independent experiments. (**b**) Representative images of erythrocyte adhesion under CTL and CS2 conditions (at a parasitemia value of 50%). Scale bar: 10 µm.

Cell adhesion to the trophoblast side of the modeled placental barrier was quantified by the percent of area coverage by the erythrocytes after injecting and incubating infected and uninfected (control) erythrocytes into trophoblast channel (Fig. 3a). Regarding the control, area coverage by erythrocytes was found to be 5.0 + 0.8%, due to nonspecific adhesion. For the IE specimens, area coverage was significantly higher, at the level of 16.1 + 0.7% (average from 3 independent experiments with parasitemia of 26%, 50% and 95%, respectively) These experiments revealed that the level of sequestration for different IE preparations did not depend on parasitemia levels due to saturation of the sequestration sites on BeWo cell layer in the employed conditions (time of incubation and washing conditions). This saturation has an advantage in that we can average results on glucose transfer measurement from different experiments in our device as the level of IE sequestration in each experiment is the same. Representative images of cell adhesion to the BeWo cells for both control and CS2 IEs are shown in Fig. 3b.

### Glucose transport across the placental barrier

The molecular concentration distribution in microchannels is a result of the balance between convection and diffusion mechanism. Because of considering fluid flows, the convection mechanism is modeled based on the incompressible Navier–Stokes equation,

1 $$\rho \left[\frac{\partial {\varvec{U}}}{\partial t}+{\varvec{U}}.\nabla {\varvec{U}}\right]=-\nabla P+\mu \Delta {\varvec{U}}+\rho {\varvec{g}}$$

where *t* is time, *ρ* is the fluid density, ***U*** is the velocity vector field, *μ* is the fluid viscosity *P* is the pressure, and ***g*** is gravity vector. As, the flow velocity in both channels is small, inertial force is neglected. The concentration distribution of given species is governed by convection–diffusion equation,

2 $$\frac{{\partial C}_{G}}{\partial t}+\left({\varvec{U}}.\nabla {C}_{G}\right)=D{\nabla }^{2}{C}_{G}$$

where *D* is the diffusion coefficient of the species.

Cells consume nutrients such as glucose to survive and proliferate. The cell consumption of glucose was modeled using the Michaelis–Menten kinetics^44^,

3 $$\frac{{\partial C}_{G}}{\partial t}=D{\nabla }^{2}{C}_{G}-\nabla .\left({\varvec{U}}.{C}_{G}\right)-\frac{{V}_{max.G }N{C}_{G}}{{K}_{mG}+{C}_{G}}$$

where the cell density and the concentration are shown by *N* (cells/ml) and *C*_*G*_, respectively. ${V}_{max.G}$ is a function of the maximum uptake rate, ${K}_{mG}$ is the Michaelis–Menten constant for HUVECs, BeWo cells, erythrocytes, and IEs. These parameters together with the total amount of cellular consumption within 2 h experiments are summarized in Table 1. The glucose consumption by the sequestered IEs is two orders of magnitude smaller than the HUVECs and BeWo cells consumptions within 2 h experiments.

**Table 1:** Glucose consumption parameters and values.

Parameter	Cell type	Value	Reference
Maximum uptake rate of glucose(${V}_{\mathrm{max}.G}$) ($\mathrm{\mu mol }{ 10}^{-6}$ cells ${\mathrm{d}}^{-1}$)	HUVECs	1.8	^61^
	BeWo	1.1	^62^
	Erythrocytes	0.005	^63^
	IEs	0.5	^63^
Michaelis–Menten constant for glucose uptake ${(\mathrm{K}}_{\mathrm{mG }},$ mM)	HUVECs	1.23	^62,64^
	BeWo	1.5	^65^
	IEs	0.97	^66^
Cellular glucose consumption in 2 h (mmol)		CTL (n = 9)	CS2 (n = 11)
	ΔC_HUVEC_	6.9 × 10^–5^	
	ΔC_BeWo_	2.9 × 10^–5^	
	ΔC_IE_	–	6.5 × 10^–7^
	ΔC_Erythrocytes_*	–	–

*Erythrocyte glucose uptake rate is two orders of magnitude lower than IEs. The cellular consumption was therefore neglected in the calculation.

### Effects of IE sequestration on glucose transport

**Figure 4:**
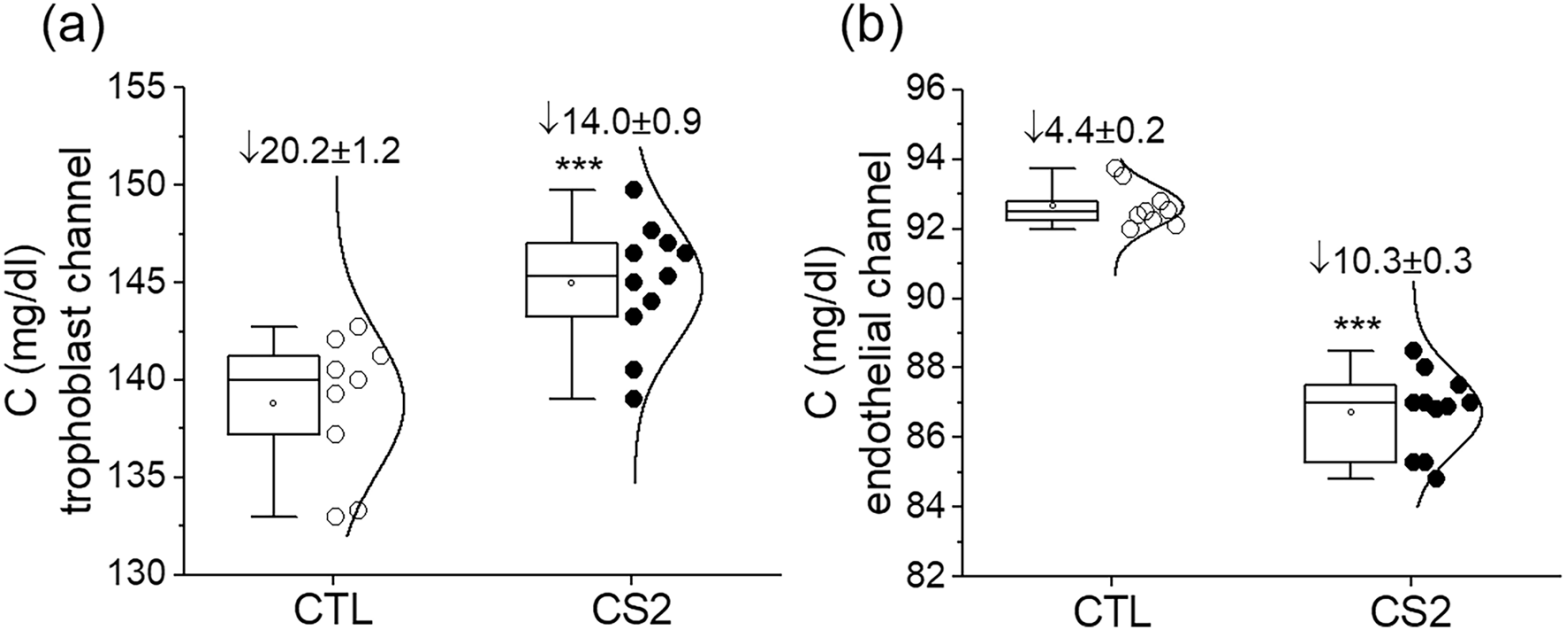
Glucose concentrations after 2 h perfusion experiments in (**a**) the trophoblast channel and (**b**) the endothelial channel. Reduction is shown relative to initial concentration at the beginning of the experiment. *** *p* < 0.001.

Effects of IE sequestration on glucose transport across the placental barrier were found to be pronounced, as measured by the glucose concentrations in the placental barrier model after 2 h perfusion. In the trophoblast channels, the difference between the uninfected erythrocytes (control) and CS2 IEs was significant (*p* < 0.001), as shown in Fig. 4a. As compared to the initial glucose concentration, glucose concentration dropped 20.2 ± 1.2 mg/dl and 14.0 ± 0.9 mg/dl for control and CS2 IEs, respectively. In the fetal circulation channels, the glucose concentration in the CS2 IEs is significantly (*p* < 0.001) lower than the control; it dropped 10.3 ± 0.3 mg/dl, as compared to only 4.4 ± 0.2 mg/dl for the control group.

**Figure 5:**
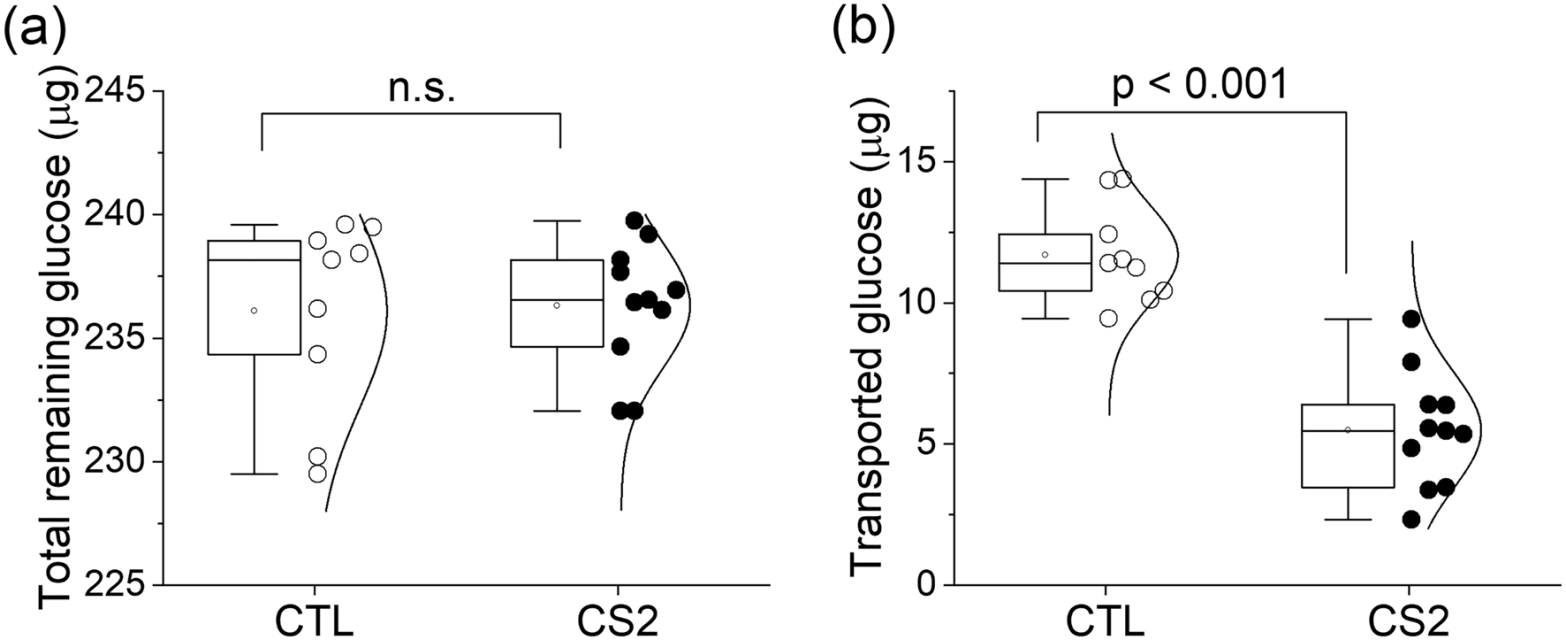
(**a**) Total glucose remained in the device, determined as the sum of glucose amount in both trophoblast and endothelial channels. n.s.—not significant. (**b**) Amount of glucose transported across the modeled placental barrier.

Figure 5 shows the total glucose remained in the placental barrier model after 2 h perfusion experiment, as the sum of glucose amount in the trophoblast and endothelial channels. The remaining glucose was found to be quite similar between the PM (CS2 IEs) and control (uninfected erythrocytes), as 236.32 ± 0.77 µg and 236.09 ± 1.31 µg, respectively. This observation agrees well with our theoretical calculation (Table 1) where the loss of glucose in the placental barrier model is primarily due to cellular consumption by HUVECs and BeWo cells and the consumption by IEs is about two orders of magnitude lower. This allowed us to further determine the effect of IE sequestration on the approximate amount of glucose transported across the placental barrier, Δm during the 2 h perfusion, by solving the equations of conservation of mass within the device,

4 $${m}_{Maternal}^{2h}={m}_{Maternal}^{0h}-{m}_{BeWo}-{m}_{IE}-\Delta m$$

5 $${m}_{Fetal}^{2h}={m}_{Fetal}^{0h}-{m}_{HUVEC}+\Delta m$$

where m is the amount of glucose, the superscript 0 h and 2 h stands for the experimental measurement of glucose, the subscripts of Maternal and Fetal stand for the trophoblast and endothelial channels of observation, and the subscripts of BeWo, IE, and HUVEC stand for the amount of glucose consumed by each cell type. Considering the glucose change in each channel, ${m}^{0}-{m}^{2h}$, can be determined by the experimental measurement of the glucose concentration (Fig. 4), and the cellular glucose consumption estimated from theoretical calculations (Table 1), the amount of glucose transported across the placental barrier, Δm was found to be 5.49 ± 0.62 µg in the CS2 IEs, which is much less than control, 11.70 ± 0.58 µg (Fig. 5b). Such drastic difference between the glucose transport between the IEs and control groups was believed to be attributed by IE sequestration and its associated factors, including the elevated resistance to glucose perfusion by blocking the mass transfer across the barrier and altered glucose permeability of the placental barrier due to CS2-BeWo interactions. Our results suggest that just IE sequestration, even without inflammation initiated by monocytes/macrophages, may play a role in the insufficient delivery of nutrients (glucose in this work) from mother to fetus during placental malaria, potentially contributing to the fetal underdevelopment and low birth weight pathologies.

#### Limitations of the model

As placenta is made up of branching tree-like structures called villous trees and a network of densely branched blood vessels appears during placenta development^31^, we think that maternal blood flow through these structures is quite different from laminar unidirectional flow (as might be the case in other parts of organism) and actually might even be better modeled by bi-directional flow at appropriate shear stresses. For example, as initially hypothesized by McLaren and Michie^45^ and later tested in mouse models by Vom Saal and Dhar^45,46^, the direction of blood flow in uterine artery is bidirectional. Therefore, we decided that bidirectional flow in our model obtained by rocking motion might not only be a very convenient way to control for precise shear stress, but also simulate bifurcations of blood flow in the vicinity of placental villous trees. Further, during an in vitro culture of BeWo and HUVEC cells for the device, this rotary motion is similar to what is commonly used for efficient cell growth when cells are placed in the rocking platform, which also provides the movement of culture media for a better gas and nutrients exchange. This is especially important for growth of the cells in small channels of the device. Moreover, our experiment with the bidirectional flow of blood (after cells grew to confluence in the channel) takes only 2 h, which is unlikely to produce any significant impact on the cell phenotype, viability, and other cell responses. Besides, we use the control with uninfected RBCs to which we compare the results obtained with IE.

The wall shear stress created by the fluid flow from the rocking motion (± 7°) was 0–1.41 dynes/cm^2^ in our model, which was physiologically relevant. Blood flow and its force are also important for native microvessels to maintain quiescent phenotype of vascular cells as well as for mammalian cell cultures. The shear stress values in physiological blood flow as seen in similar sized small veins^47^ range in 1–6 dynes/cm^2^. Importantly, maintaining low shear stress at the maternal–fetal interface has been demonstrated to be important in promoting maximal exchange of nutrients and waste^48^. Detailed shear stress levels that syncytiotrophoblast layer is experiencing in vivo are still not well understood. However, according to a computational model of placenta^49^, the shear stress exerted to the syncytiotrophoblast in the intervillous space is highly heterogeneous, varying from 0.5 ± 0.2 to 2.3 ± 1.1 dynes/cm^2^. The shear stress level in the placental microvasculature^50^ was found to be 0.6 dynes/cm^2^ under normal conditions, which can increase to 1 dynes/cm^2^ and higher under pathological conditions.

In this study, type I collagen gel (with pore sizes of 2–3 µm^51,52^) was used as a surrogate for villous stroma. In literature, both type I and type IV collagen have been used to create in vitro placental barrier models^53–56^. Immunohistochemical analysis also showed these two collagen types are expressed but with different quantifications in varied locations (e.g., intima, media, perivascular area, and triangular area) in villous stroma of stem villi^57^. Collagen type I was also chosen for good cell attachment. It is a limitation of current model in that this collagen membrane is different to some extent from the natural membrane. As glucose molecules have much smaller size than the pores in the natural basement membrane or in ECM^58,59^ , the actual barrier for their transport is the two layers of epithelial and endothelial cells on both sides of the membrane ^60^.

#### Conclusions

In this paper, we have reported a placenta-on-a-chip device to model and study the pathological events in placental malaria. A microfluidic 3D cell culture platform with HUVECs and BeWo cell cultured in two microchannels on both sides of ECM gel were used to mimic the placental barrier in vitro. The permeability of the placental barrier for glucose was analyzed to compare effects of presence of CSA-adherent malaria-infected and uninfected erythrocytes. The results demonstrated that CSA-binding IEs added resistance to the placental barrier for glucose perfusion and decreased the glucose transport across the placental barrier.

## Data Availability

The original data in this study are available by contacting the corresponding author.
